# Genetically Engineered Resveratrol-Enriched Rice Inhibits Neuroinflammation in Lipopolysaccharide-Activated BV2 Microglia Via Downregulating Mitogen-Activated Protein Kinase-Nuclear Factor Kappa B Signaling Pathway

**DOI:** 10.1155/2018/8092713

**Published:** 2018-12-03

**Authors:** Lalita Subedi, So-Hyeon Baek, Sun Yeou Kim

**Affiliations:** ^1^College of Pharmacy, Gachon University, #191 Hambakmoero, Yeonsu-gu, Incheon 21936, Republic of Korea; ^2^Department of Well-being Resources, Sunchon National University, Sunchon, Jeonnam 57922, Republic of Korea

## Abstract

Resveratrol, a natural stilbenoid, is produced by several plants, especially grape vines. Its strong potency against obesity, metabolic disorders, vascular disease, inflammation, and various cancers has already been reported. Large amounts of wine or grapes need to be consumed to obtain the amount of resveratrol required for biological activity. Pure resveratrol at concentrations as low as 10 *μ*M induces cytotoxicity to normal cells. To overcome these limitations, we prepared genetically modified resveratrol-enriched rice (RR). We previously reported the strong antiaging potential of RR against ultraviolet B/reactive oxygen species-induced toxicity in normal human dermal fibroblasts (NHDF). As aging is characterized by neuroinflammation and neurodegeneration, we further evaluated the role of RR against LPS-induced neuroinflammation. RR inhibited nitric oxide production and the expression of inflammatory proteins such as iNOS and COX-2. RR significantly modulated mitogen-activated protein kinase signaling, activator protein AP-1 signaling, and nuclear factor kappa B (NF-*κ*B) mediated transcription of inflammatory proteins via inhibition of NF-*κ*B translocation, IkB phosphorylation, and proinflammatory cytokine productions such as interleukin IL-6, IL-1*β*, tumor necrosis factor alpha (TNF-*α*), and prostaglandin E2 (PGE2). These findings show that the strong antineuroinflammatory effects of RR can be beneficial for aging-mediated neurodegenerative conditions as well as disorders of the central nervous system caused by neuroinflammation.

## 1. Introduction

Inflammation is the major cause and aggravating factor of various acute or chronic pathological conditions, including photoaging, diabetes, and cancer [1, 2]. Similarly, neuroinflammation is the key mediator of secondary brain damage in most of the neurological disorders, such as Alzheimer's disease (AD), Prion disease, Parkinson's disease (PD), multiple sclerosis (MS), ischemic stroke, experimental autoimmune encephalomyelitis (EAE), and neuropathic pain [3–5]. Neuroinflammation is induced by aging-dependent conditions and aging-independent pathological events, which share similar inflammatory cascades [6–8]. Microglia, neurons, astrocytes, and oligodendrocytes are the basic cells of the brain. Microglia and astrocytes, as glial cells, have a role to defend against brain injury to maintain homeostasis and repair brain injury. In aging-dependent conditions and aging-independent disorders such as AD, PD, and stroke, neuroinflammation can be initiated by chronic microglial activation. Activated microglia are required for basic immune defense in the brain; however, chronic microglial activation is toxic to the central nervous system (CNS) [9]. Conversion of normal microglia to toxic microglial M1 phenotype is responsible for the initiation of inflammation in the CNS through the production of reactive oxygen species (ROS), nitric oxide (NO), proteases, arachidonic acids, excitatory amino acids, and cytokines [10]. These neurotoxic substances are responsible for the disruption of architecture and function of neurons, synaptic degeneration, neuronal loss, and ultimately neurodegeneration [11]. The production of ROS and other inflammatory mediators and oxidative stress are closely related to mitogen-activated protein kinase (MAPK) signaling, as well as nuclear factor kappa B (NF-*κ*B) and activator protein- (AP-) 1-mediated transcription [12–16]. Hence, natural compounds or nutraceuticals with the potential to regulate these steps to control microglial activation will be promising candidates for inhibiting neuroinflammation and neurodegenerative conditions. Although advances have been made to treat such neuroinflammatory conditions, there is a lack of effective therapeutic strategy to cure these disease conditions. In the past few decades, there has been a growing interest toward alternative medicines, especially phytochemicals, as therapeutic agents for neurological disorders involving activated microglia-mediated neuroinflammation [17–20].

Resveratrol has been studied for decades for its multifunctional potency against many human ailments [21], including neuroinflammatory conditions [22, 23]. Resveratrol is widely present in a number of grape species, berries, peanuts, soy, and red wine [24]; however, we have to consume a large amount of these foods to get the sufficient amount of resveratrol required for its biological activity [25]. Additionally, the cytotoxic nature of resveratrol on normal cells overshadows its extensive potential against human ailments [26]. To overcome this limitation and achieve therapeutic potential, resveratrol-enriched rice (RR) was developed by taking advantage of genetic engineering [27].

In this study, we developed RR through genetic engineering techniques, as described previously [27]. Rice is a main food component among Asian population, and therefore, rice consumption is higher than other food components. Resveratrol biosynthesis gene was transcribed into normal dongjin rice (NR) to produce RR. We previously reported that RR has a better potency to inhibit ultraviolet B (UVB)/ROS-induced aging by maintaining matrix metalloproteinase (MMP1)/procollagen I (PIP) balance an inhibiting inflammation and apoptosis, without any cellular toxicity [28].

RR can be used safely and more effectively in cases where resveratrol shows efficacy to treat or control neuroinflammatory conditions. Therefore, in this study, we compared the efficacy of NR, RR, and resveratrol in terms of cytotoxicity and anti-inflammatory potential in activated microglia and elucidated the possible mechanisms underlying the antineuroinflammatory potential of RR in lipopolysaccharide- (LPS-) stimulated BV2 murine microglial cells. We hope our research clearly revealed the additive role of RR from NR and resveratrol which could be the best alternative treatment for the neuronal disorders induced by neuroinflammation like AD, PD, and MS lesions.

## 2. Materials and Methods

### 2.1. Materials

Dulbecco's modified Eagle medium (DMEM), fetal bovine serum (FBS), and penicillin-streptomycin were purchased from Invitrogen (Carlsbad, CA, USA). LPS, NG-mono-methyl-L-arginine (L-NMMA), and trans-resveratrol were purchased from Sigma-Aldrich (St. Louis, MO, USA). A normal rice (NR) (*Oryza sativa* var. japonica) and resveratrol-enriched rice (RR) were supplied by the Rural Development Administration (RDA) of South Korea as mentioned in our previous paper [28]. Enzyme-linked immunosorbent assay (ELISA) development kits for tumor necrosis factor alpha (TNF-*α*), interleukin-6 (IL-6), prostaglandin E2 (PGE2), and IL-1*β* were purchased from R&D Systems (Minneapolis, MN, USA). Primary and secondary antibodies against inducible nitric oxide synthase (iNOS), cyclooxygenase (COX-2), extracellular signal-regulated kinases (ERK), C-Jun N-terminal kinase (JNK), p38, NF-*κ*B, Histone-3, *β*-Actin, IkB, pIkB, C-Fos, p-C-Fos, C-Jun, p-C-Jun, and tubulin were purchased from Cell Signaling (Beverly, MA, USA). All other chemicals and reagents were purchased from Sigma Chemical (St. Louis, MO).

### 2.2. NR and RR Extraction

NR and RR received from RDA were extracted in methanol (MeOH). The rice grains (10 g) were incubated with 100 mL of MeOH for 60 min. During this period, the extraction mixture was placed in an ultrasonic water bath with sonication. After 60 min incubation of the extraction mixture with sonication, the mixture was filtered (HYUNAI Micro N0.20 filter paper, Korea) and then evaporated using a rotary evaporator at 40°C for the removal of methanol. The evaporated extract yield was further freeze-dried for complete lyophilization of the extract. The final yield of the extract powder was stored and used for experiment. A stock solution (100 mg/mL) was prepared in dimethyl sulfoxide (DMSO). This stock solution was diluted in DMEM and used for cell treatment during experiments.

### 2.3. Cell Culture

BV-2 murine microglia were used to study the anti-inflammatory effects of NR, RR, and resveratrol. The BV-2 microglial cell lines were obtained as gift samples from Dr. E. Choi, Korea University (Seoul, Korea). BV2 cells were maintained in DMEM supplemented with 10% heat-inactivated FBS, penicillin (1 × 10^5^ U/L), and streptomycin (100 mg/L) in a humidified incubator with 5% CO_2_ at 37°C.

### 2.4. Cell Treatment and Cell Viability Assay

The effect of NR, RR, and resveratrol on the cytotoxicity of BV2 cells was measured using 3-(4,5-dimethylthiazol-2-yl)-2,5-diphenyl-tetrazolium bromide (MTT) assay. The cells cultured in 96-well plates were treated with different concentrations of samples (NR, RR, and resveratrol) with or without LPS. In the pretreatment condition, the samples were pretreated in the seeded plates, and LPS (100 ng/mL) was added 30 min after sample treatment. The cells were incubated for 24 h after LPS activation for nitrite assay and cell viability assay. After incubation for 24 h, the media were removed and MTT solution was added to the cells at a final concentration of 0.5 mg/mL. After an additional 1 h incubation, the media were carefully removed and 200 *μ*L of DMSO was added to each well. The optical density (OD) was measured on a plate reader at 570 nm. Cell viability was evaluated by observing the ability of viable cells to reduce yellow-colored MTT to purple-colored formazan. The results were expressed as the percentage of LPS-treated group (LPS-treated cells).

### 2.5. Nitric Oxide Assay

The inhibitory effect of NR, RR, and resveratrol on NO synthesis was determined using BV-2 microglial cells activated with 100 ng/mL LPS. BV2 cells were seeded at a density of 4 × 10^4^ cells/well in a 96-well plate 24 h before treatment. The seeded cells were treated with NR, RR, and resveratrol the next day. The cells were activated with LPS after 30 min [9] and further incubated for 24 h. After 24 h incubation, the nitrite level was measured in the culture media using Griess reagent (1% sulfanilamide and 0.1% N-1-napthylethylenediamine dihydrochloride in 5% phosphoric acid). The conditioned medium (50 *μ*L) was transferred to a new 96-well plate and mixed with an equal volume of Griess reagent. This mixture gives a pink color because of the presence of NO. The colorimetric change was quantified by measuring the OD of the solution in 96-well plates using a microplate reader at 540 nm. NG-mono-methyl-L-arginine (L-NMMA), a well-known NOS inhibitor [10], was used as a positive control. NaNO_2_ was used as the standard to compare the amount of nitrite in the conditioned medium. Acute microglial activation was performed by LPS treatment for 6 h, and chronic microglial activation was performed by LPS treatment for 24 h.

### 2.6. Measurement of PGE2, TNF-*α*, IL-1*β*, and IL-6 Production

BV2 microglial cells were treated with the samples (NR, RR, and R) and activated with LPS to measure TNF-*α*, IL-1*β*, PGE2, and IL-6 levels under neuroinflammatory conditions. BV2 cells were seeded at a density of 1.5 × 10^6^ cells/well in DMEM and incubated for 24 h. The seeded cells were treated with the samples, followed by LPS treatment for microglial activation, and incubated for 24 h. After 24 h of incubation, conditioned medium from the treated plate was collected and used for measuring the levels of PGE2, TNF-*α*, IL-1*β*, and IL-6. The collected conditioned medium can be stored at −20°C until later use. PGE2 was measured using a competitive enzyme immunoassay kit (Cayman Chemical, Ann Arbor, MI, USA). TNF-*α*, IL-1*β*, and IL-6 levels were measured using ELISA development kits (R&D Systems, Minneapolis, MN, USA).

### 2.7. NF-*κ*B Assay

BV2 cells were seeded at a density of 1.5 × 10^6^ cells/well in a 6-well plate and treated with NR, RR, and resveratrol, followed by LPS treatment for microglial activation and NF-kB translocation and transcription. The cells treated with the samples and LPS were incubated for 1 h and then harvested. Nuclear and cytosolic extracts from the harvested microglial cells were prepared using a Nuclear/Cytosolic Extraction Kit (Active Motif, Carlsbad, CA) according to the manufacturer's protocol. The protein levels of NF-*κ*B, histone-3, IkB, and p-IkB were evaluated by Western blot analysis. The expression of nucleolar and cytosolic NF-*κ*B was measured using histone-3 and *β*-actin as loading controls, respectively. The expression of IkB and p-IkB was observed in the cytosolic fraction. The absence of *β*-actin expression in the nuclear fraction suggests the clear separation of nuclear and cytosolic fractions during fractionation without any contamination. Densitometry analysis of the bands was performed using ImageMaster™ 2D Elite software (version 3.1, Amersham Pharmacia Biotech, Buckinghamshire, UK).

### 2.8. Western Blot Analysis

BV-2 cells were seeded at a density of 6 × 10^5^ cells/well in a 6-well plate and treated with the samples. LPS was incubated for the desired period based on the protein activation pattern according to target protein location in the cells. The cells were harvested with ice-cold phosphate-buffered saline (PBS) and centrifuged at 7500 rpm for 5 min. PBS was removed, and the cell pellets were lysed with lysis buffer [50 mM Tris-HCl (pH 8.0), 0.1% sodium dodecyl sulfate (SDS), 150 mM NaCl, 1% NP-40, 0.02% sodium azide, 0.5% sodium deoxycholate, 100 *μ*g/mL phenylmethylsulfonyl fluoride (PMSF), and 1 g/mL approtinin] [29]. This mixture was incubated in ice for 2 h. The cell lysate/protein extract was obtained after ultracentrifugation of the cell and lysis buffer mixture at 12,000 rpm for 30 min at 4°C. Total proteins (30 *μ*g) from each group were separated by 10% SDS-polyacrylamide gel electrophoresis (PAGE), transferred to nitrocellulose membranes, and incubated with primary antibodies against tubulin, iNOS, COX-2, ERK, pERK, JNK, pJNK, p38, pP38, NF-*κ*B, histone-3, *β*-actin, IkB, pIkB, C-Fos, p-C-Fos, C-Jun, p-C-Jun, and *α*-tubulin. The membranes were incubated with horseradish peroxidase-conjugated secondary antibodies, and the protein bands were visualized using ECL Western blotting detection reagents (Amersham Pharmacia Biotech). Densitometry analysis of the bands was performed using ImageMaster™ 2D Elite software (version 3.1, Amersham Pharmacia Biotech).

### 2.9. Statistical Analysis

The results were evaluated using the Statistical Analysis System (GraphPad Prism 5, La Jolla, CA, USA). The results are presented as mean ± standard error of the mean (SEM), and all results are the mean of at least three independent experiments. A statistical comparison of different treatment groups was performed by one-way analysis of variance (ANOVA), followed by Newman-Keuls multiple comparison test. A value of *p* < 0.05 was considered statistically significant.

## 3. Results

### 3.1. NR, RR, and Resveratrol Significantly Inhibit Nitrite Production in LPS-Activated Microglia

**Figure 1:**
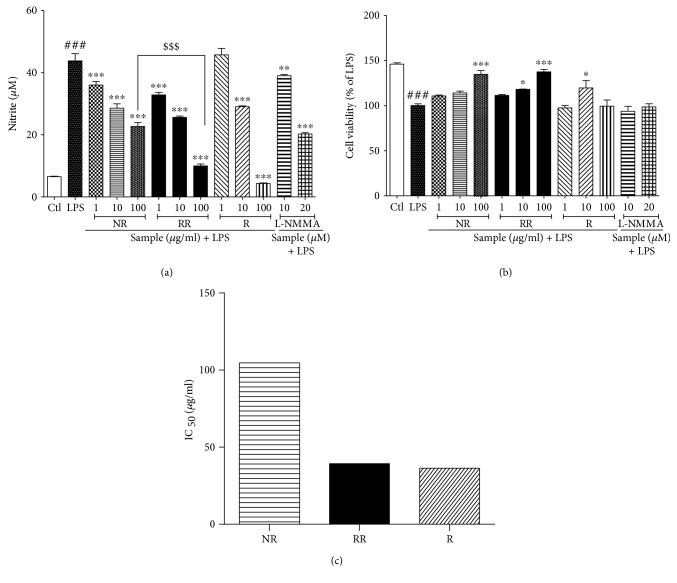
Treatment with resveratrol-enriched rice inhibits nitrite production in lipopolysaccharide-activated BV2 microglial cells without cellular toxicity. BV2 microglial cells were pretreated with normal rice (NR), resveratrol-enriched rice (RR), and resveratrol after 30 min of LPS (100 ng/mL) stimulation. (a, b) Nitrite production and cell viability after NR, RR, and resveratrol treatment. (c) IC_50_ value of NR, RR, and resveratrol samples. The concentration of samples was given in *μ*M. All data are presented as the mean ± standard error of the mean of three independent experiments. ^∗^*P* < 0.05, ^∗∗^*P* < 0.01, and ^∗∗∗^*P* < 0.001 indicate significant differences compared with LPS treatment alone. #*P* < 0.05, ##*P* < 0.01, and ###*P* < 0.001 indicate significant differences compared with untreated control group. $$$*P* < 0.001 indicate significant differences to RR compared to NR. Ctl, untreated control and LPS, lipopolysaccharide.

NO production is an important biomarker for almost all types of inflammation, especially LPS-induced neuroinflammation. We evaluated the nitrite oxide level of NR, RR, and resveratrol with L-NMMA as the positive control against LPS-activated BV2 microglial NO production. NR and RR showed the highest potency to inhibit nitrite production. Also, a resveratrol showed excellent potency at a high concentration (100 *μ*g/mL). However, NR and RR showed better potency than resveratrol at concentrations of 1 and 10 *μ*g/mL. Although RR extract has lesser amount of pure resveratrol than resveratrol, it has higher potency with lower IC_50_ value, suggesting that RR does not cause cellular toxicity. The effect of RR (100 *μ*g/mL) on nitrite production is statistically significant to that of NR alone suggesting that RR has better potency than that of the NR. NR and RR treatment protected against LPS-induced toxicity, especially up to a concentration 100 *μ*g/mL, by increasing the number of viable cells. In case of resveratrol treatment, only 10 *μ*g/mL protected against LPS toxicity by increasing the viable cells, but not 100 *μ*g/mL. NR and RR treatment at concentrations of 1 *μ*g/mL and 10 *μ*g/mL demonstrated higher potency in inhibiting nitrite production than L-NMMA, suggesting that NR and RR are better alternatives than L-NMMA for nitrite production inhibition as shown in (Figure 1).

#### 3.2. RR Treatment Inhibits the Expression of iNOS and COX-2 in Acute and Chronic LPS Activation

**Figure 2:**
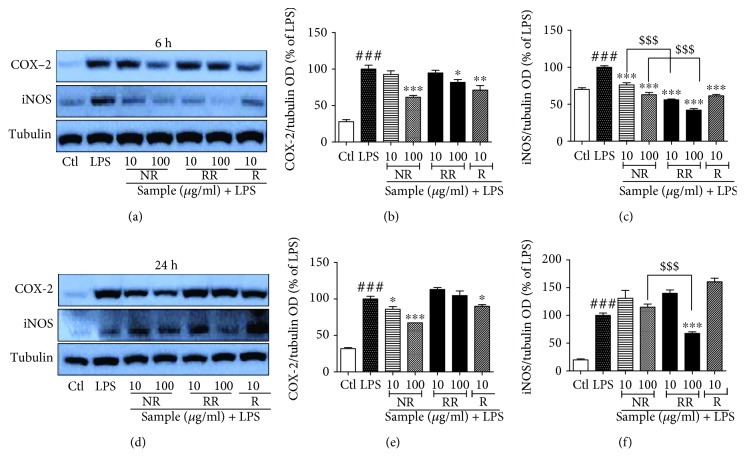
Treatment with resveratrol-enriched rice inhibits the expression of iNOS and COX-2 in lipopolysaccharide-activated (acute and chronic) BV2 microglial cells. BV2 microglial cells were pretreated with normal rice (NR), resveratrol-enriched rice (RR), and resveratrol after 30 min of LPS (100 ng/mL) stimulation. (a–c) iNOS and COX-2 expression and their band intensity in LPS-activated BV2 microglial cells after 6 h of sample treatment and LPS activation. (d–f) iNOS and COX-2 expression and their band intensity in LPS-activated BV2 microglial cells after 24 h of sample treatment and LPS activation. Tubulin was used as loading control. All data are presented as the mean ± standard error of the mean of three independent experiments. ^∗^*P* < 0.05, ^∗∗^*P* < 0.01, and ^∗∗∗^*P* < 0.001 indicate significant differences compared with LPS treatment alone. ###*P* < 0.001 indicates significant differences compared with untreated control group. $$$*P* < 0.001 indicates significant differences to RR compared to NR. Ctl, untreated control and LPS, lipopolysaccharide.

As iNOS and COX-2 proteins are precursors of NO production and inflammatory cascades, we further evaluated the role of NR and RR in regulating the expression of iNOS and COX-2 in LPS-activated BV2 microglial cells for acute as well as chronic activation conditions. LPS treatment in BV2 cells significantly increased the expression of iNOS and COX-2. We performed acute microglial activation, which is the incubation of cells with a compound and LPS for 6 h, to determine the altered expression of iNOS and COX-2 in LPS-activated BV2 microglial cells. We also performed incubation for 24 h, which is defined as chronic microglial activation, and evaluated the expression of iNOS and COX-2. We found that NR and resveratrol showed a high potency to inhibit COX-2 expression in acute and chronic activation conditions. When compared with NR and R, RR demonstrated poor but significant efficacy in acute activation, but no significant activity in chronic activation. In contrast, RR exhibited the highest potency to inhibit iNOS expression in both acute and chronic activation conditions. NR and resveratrol did not demonstrate significant inhibition of iNOS in chronic activation condition, while a significant potency was observed in acute activation condition as shown in (Figure 2). The effect of RR on inhibition of iNOS expression is statistically significant in comparison to the NR. This result suggests that additive effect of NR and R on RR is more significant than that on NR and R alone for the inhibition of iNOS expression. We hypothesized that the potent iNOS/NO inhibiting ability of RR might be responsible for its anti-inflammatory effects.

#### 3.3. Bioactive-Phytochemicals from NR, Namely, *α*-Tocopherol and *γ*-Tocopherol, Inhibit the Nitrite Production and Expression of iNOS and COX-2 against LPS-Activated Microglia

**Figure 3:**
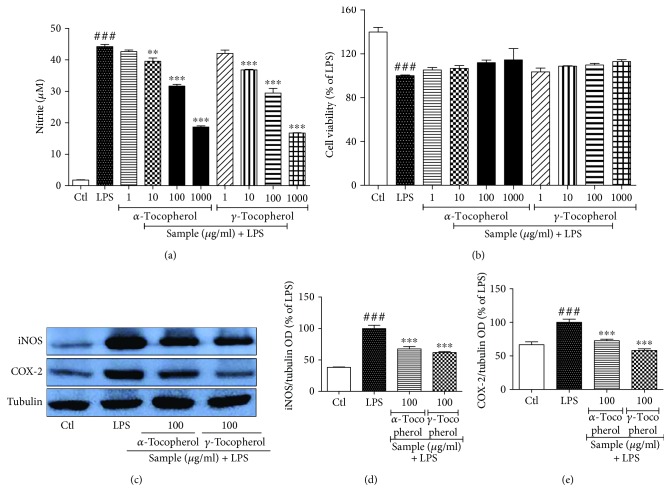
Treatment *α*-tocopherol and *γ*-tocopherol inhibits the nitrite production and expression of iNOS and COX-2 against LPS-activated microglia cells. BV2 microglial cells were activated with LPS (100 ng/mL) 30 min before sample treatment, and samples of different concentrations were added under the same condition. Incubation of LPS and sample was performed for 6 h for iNOS and COX-2 expression and 24 h for nitrite production assay. (a, b) Nitrite production and cell viability of LPS-activated BV2 microglial cells after treatment of *α*-tocopherol and *γ*-tocopherol (c–e) iNOS and COX-2 expression and their respective band intensity. Tubulin was used as loading control. All data are presented as the mean ± standard error of the mean of three independent experiments. ^∗∗^*P* < 0.01 and ^∗∗∗^*P* < 0.001 indicate significant differences compared with LPS treatment alone. ###*P* < 0.001 indicates significant differences compared with untreated control group. Ctl, untreated control and LPS, lipopolysaccharide.

We expected that the safety and compatibility of normal rice and the resveratrol together can show the similar potency of RR against neuroinflammation. However, interestingly, normal rice alone also showed the antineuroinflammatory effect against LPS-treated BV2 cells. To further confirm this effect and to find the responsible bioactive phytochemical in NR, we selected *α*-tocopherol and *γ*-tocopherol to evaluate its role against neuroinflammation in LPS-treated BV2 cells. We observed that treatment of the *α*-tocopherol and *γ*-tocopherol significantly inhibited the production of nitrite in LPS-activated microglia. As we treated the sample of 1, 10, 100, and 1000 *μ*g/mL concentration, potency of sample shows concentration-dependent manner and did not show any cellular toxicity as evidenced by cell viability assay. Although *γ*-tocopherol has been reported to inhibit LPS-induced macrophage cell toxicity previously [30], here, we found the similar potency of *α*-tocopherol and *γ*-tocopherol to inhibit the inflammatory mediators against LPS-activated microglia as shown in Figure 3.

#### 3.4. NR, RR, and R Can Modulate MAPK Signaling in Acute LPS-Activated BV2 Microglial Cells

**Figure 4:**
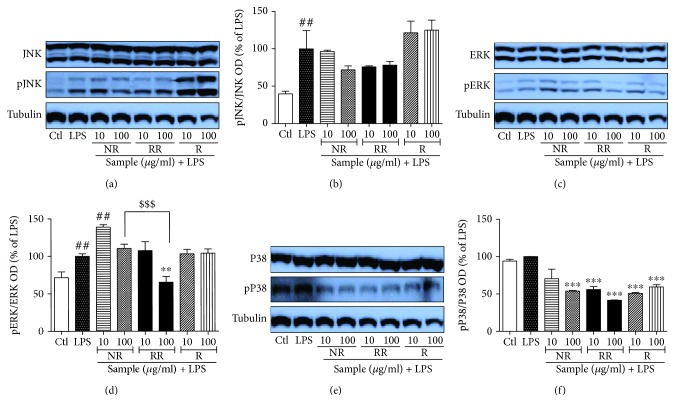
Treatment with resveratrol-enriched rice modulates MAPK signaling in lipopolysaccharide-activated (acute) BV2 microglial cells. BV2 microglial cells were activated with LPS (100 ng/mL) 30 min before sample treatment, and samples of different concentrations were added under the same condition. Incubation of LPS and sample was performed for 30 min. MAPK expression was measured in pretreatment in LPS-activated BV2 cells. (a, b) JNK/pJNK expression and band intensity. (c, d) ERK/pERK expression and band intensity. (e, f) p38/pP38 expression and band intensity in LPS-activated BV2 microglia. All data are presented as the mean ± standard error of the mean of three independent experiments. ^∗^*P* < 0.05, ^∗∗^*P* < 0.01, and ^∗∗∗^*P* < 0.001 indicate significant differences compared with LPS treatment alone. ##*P* < 0.01 indicates significant differences compared with untreated control group. $$$*P* < 0.001 indicates significant differences to RR compared to NR. Ctl, untreated control and LPS, lipopolysaccharide.

MAPKs are activated by phosphorylation of tyrosine and threonine residues, which in turn leads to a signaling cascade that upregulates the production of inflammatory mediators as well as proinflammatory cytokines in activated microglia or under other inflammatory conditions. Therefore, we evaluated the role of NR and RR in altering the activation/phosphorylation of MAPK proteins (p38, JNK, and ERK). LPS-activated microglia showed significantly increased phosphorylation of JNK, ERK, and P38, which might be responsible for the induction of transcription of inflammatory mediators. Treatment of LPS-activated BV2 cells with NR and RR significantly reduced expression of phosphorylated JNK and P38. In case of ERK phosphorylation, only RR showed high efficacy, while NR did not demonstrate significant inhibition. The effect of RR is statistically significant in both the treated concentration to inhibit the ERK phosphorylation in comparison to the NR alone. This data suggested that additive effect of NR and resveratrol in RR is more significant than in NR and R alone. Resveratrol showed slightly different pattern in the MAPK modulation where it showed increase of JNK phosphorylation although it is not significant. Also, it did not show any changes in ERK phosphorylation whereas it significantly inhibited the p38 phosphorylation. NR and RR exhibited high potency to inhibit P38 phosphorylation than that of the ERK and JNK as shown in (Figure 4). Among all, RR showed the highest potency to inhibit the phosphorylation of all the MAPK's specially at 100 *μ*g/mL. These findings suggest that RR treatment inhibits pJNK, pERK, and pP38 nonspecifically and its potency is higher than NR and resveratrol itself.

#### 3.5. NR, RR, and R Modulate AP-1 Signaling in LPS-Activated BV2 Microglial Cells

**Figure 5:**
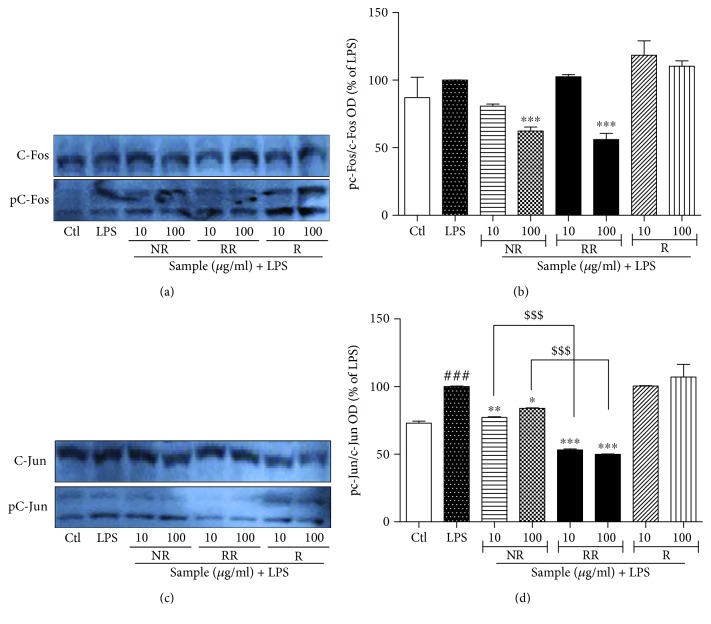
Resveratrol-enriched rice modulates AP-1 effector signaling in lipopolysaccharide-activated BV2 microglial cells. BV2 microglial cells were treated with samples, followed by LPS (100 ng/mL) activation. AP-1 signaling was evaluated after incubation of cells for 1 h. AP-1 expression was measured pretreatment in LPS-activated BV2 cells. (a, b) p-C-Fos/C-Fos protein expression and band intensity, and (c, d) p-C-Jun/c-Jun expression and band intensity after incubation with LPS and samples for 1 h in BV2 cells. All data are presented as the mean ± standard error of the mean of three independent experiments. ^∗^*P* < 0.05, ^∗∗^*P* < 0.01, and ^∗∗∗^*P* < 0.001 indicate significant differences compared with LPS treatment alone. ##*P* < 0.01 and ###*P* < 0.001 indicate significant differences compared with untreated control group. $$$*P* < 0.001 indicates significant differences to RR compared to NR. Ctl, untreated control and LPS, lipopolysaccharide.

Next, we assessed the expression of AP-1 proteins. Toxicant-induced activation of MAPK is responsible for the activation of AP-1 (C-Fos and C-Jun) in inflammatory response and other conditions [31]. In this study, we observed that the significant inhibition of pERK, pJNK, and pP38 by RR treatment was further confirmed by the downregulated expression of p-C-Fos and p-C-Jun under the similar treatment conditions as that for NF-*κ*B. NR and RR treatment at a concentration of 100 *μ*g/mL inhibited the expression of p-C-Fos where NR and RR significantly inhibited the expression of p-C-Jun at both the treated concentrations (10 and 100 *μ*g/mL). The effect of RR is statistically significant on both the treated concentration in comparison to the NR alone suggesting that additive effect of NR and resveratrol in RR is more significant than the NR and R alone. P38, JNK, and ERK activation is required for C-Jun activation. RR treatment downregulated p-C-Jun expression, indicating its strong anti-inflammatory effects against activated microglia as shown in Figure 5. As resveratrol did not significantly change the JNK and ERK phosphorylation, that result was further supported by the unchanged phosphorylation of C-Fos and C-Jun after resveratrol treatment. This data supported the fact that C-Jun and C-Fos phosphorylation were correlated with JNK and ERK phosphorylation.

#### 3.6. NR, RR, and Resveratrol Modulate NF-*κ*B Translocation in LPS-Activated BV2 Microglial Cells

**Figure 6:**
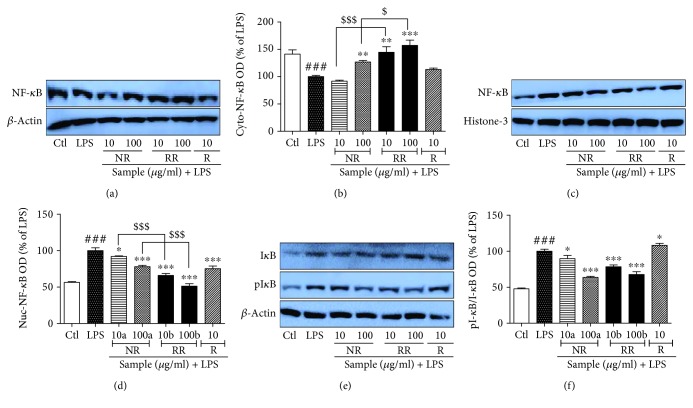
Treatment with resveratrol-enriched rice inhibits NF-*κ*B translocation and I-kB phosphorylation in lipopolysaccharide-activated BV2 cells. BV2 microglial cells were pretreated with normal rice (NR), resveratrol-enriched rice (RR), and resveratrol after 30 min of LPS (100 ng/mL) stimulation. NF-*κ*B and I-kB/pI-kB expression was determined after 1 h of LPS activation. (a, b) Cytosolic NF-*κ*B expression and band intensity. *β*-Actin was used as loading control. (c, d) Nucleolar NF-*κ*B expression and band intensity. Histone-3 was used as loading control. (e, f) Cytosolic I-kB and pI-kB expression and band intensity. *β*-Actin was used as loading control. All data are presented as the mean ± standard error of the mean of three independent experiments. ^∗^*P* < 0.05, ^∗∗^*P* < 0.01, and ^∗∗∗^*P* < 0.001 indicate significant differences compared with LPS treatment alone. #*P* < 0.05 and ###*P* < 0.001 indicate significant differences compared with untreated control group. $*P* < 0.05 and $$$*P* < 0.001 indicate significant differences to RR compared to NR. Ctl, untreated control and LPS, lipopolysaccharide.

Transcription factors play an important role in the increased production of inflammatory proteins and proinflammatory cytokines. NF-*κ*B and AP-1 play the key roles as transcription factors under inflammatory conditions. In this study, we evaluated the role of NR, RR, and resveratrol against activated microglia-induced NF-*κ*B translocation and I-kB phosphorylation. LPS treatment to BV2 cells significantly increased the translocation of NF-*κ*B from the cytosol to the nucleus, as well as the phosphorylation of I-kB. These events together can increase transcription. However, NR, RR, and resveratrol treatment reversed these events. LPS-activated BV2 cells treated with RR showed the highest expression of cytosolic NF-*κ*B and the lowest expression of nucleolar NF-*κ*B. RR treatment significantly increased the cytosolic NF-*κ*B and decreased the nuclear one which is statistically significant to that of NR-treated group as shown in Figure 6, suggesting the better potency of RR to that of NR alone. The inhibition of translocation was further supported by the significant inhibition of phosphorylated I-kB as shown in (Figure 6). In all these cases, RR demonstrated the highest potency at both the treated concentrations, suggesting its anti-inflammatory effect via inhibition of NF-*κ*B translocation and NF-*κ*B/I-kB-mediated transcription of inflammatory proteins.

#### 3.7. NR and RR Can Modulate MAPK Signaling in Chronic LPS-Activated BV2 Microglial Cells

**Figure 7:**
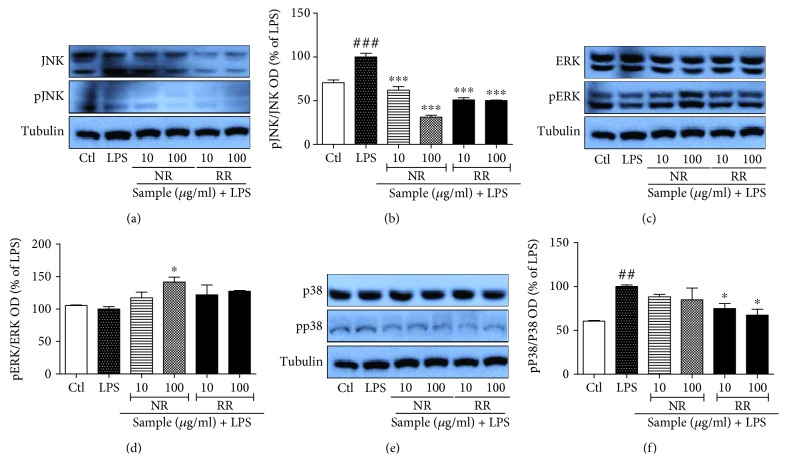
Treatment with resveratrol-enriched rice modulates MAPK signaling lipopolysaccharide-activated BV2 cells. BV2 microglial cells were pretreated with normal rice (NR), resveratrol-enriched rice (RR), and resveratrol 30 min prior to LPS (100 ng/mL) treatment. MAPK modulation was observed after 24 h of sample treatment and LPS activation. (a, b) JNK/pJNK expression and band intensity, (c, d) ERK/pERK expression and band intensity, (e, f) p38/pP38 expression and band intensity in LPS-activated BV2 microglia. All data are presented as the mean ± standard error of the mean of three independent experiments. ^∗^*P* < 0.05, and ^∗∗∗^*P* < 0.001 indicate significant differences compared with LPS treatment alone. ##*P* < 0.01, and ###*P* < 0.001 indicate significant differences compared with the untreated control group. Ctl, untreated control and LPS, lipopolysaccharide.

We evaluated the role of NR, RR, and resveratrol in altering the activation/phosphorylation of MAPK proteins (p38, JNK, and ERK) under chronic (24 h) LPS activation condition in BV2 cells. Inhibition of pJNK by NR and RR was more significant in chronic activation than in acute activation. None of the samples inhibited ERK phosphorylation in chronic activation condition, rather NR treatment significantly increased pERK expression that might suggest for its cell survival capacity against chronic activation of microglia. RR treatment also showed a slight increase in pERK expression, but it was not significant when compared with LPS-only-treated group. In case of P38 phosphorylation, although all the treated samples inhibited pP38 expression, only RR treatment showed significant inhibition when compared with LPS-only-treated group as shown in (Figure 7).

#### 3.8. NR, RR, and Resveratrol Treatment Inhibits Production of Proinflammatory Cytokines

**Figure 8:**
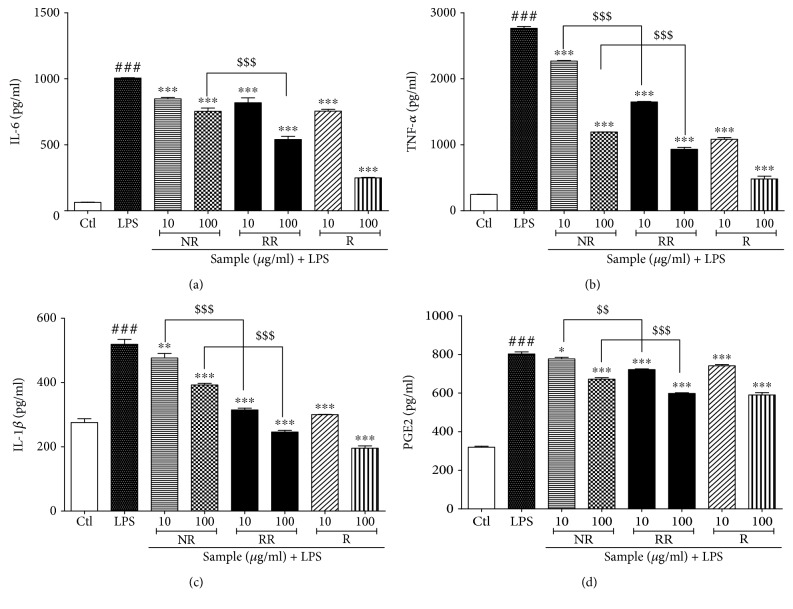
Treatment with resveratrol-enriched rice inhibits proinflammatory cytokine production in lipopolysaccharide-activated BV2 cells. BV2 microglial cells were pretreated with normal rice (NR), resveratrol-enriched rice (RR), and resveratrol 30 min prior to lipopolysaccharide (LPS; 100 ng/mL) stimulation. Proinflammatory cytokine levels were measured in the conditioned medium of treated cells using ELISA assay after 24 h of LPS activation. The proinflammatory cytokine levels in NR-, RR-, and resveratrol-treated BV2 cells were evaluated. (a) IL-6 production, (b) TNF-*α* secretion, (c) IL-1*β* secretion, and (d) PGE2 secretion. All data are presented as the mean ± standard error of the mean of three independent experiments. ^∗^*P* < 0.05, ^∗∗^*P* < 0.01, and ^∗∗∗^*P* < 0.001 indicate significant differences compared with LPS treatment alone. ###*P* < 0.001 indicates significant differences compared with untreated control group. $$*P* < 0.01 and $$$*P* < 0.001 indicate significant differences in RR compared with NR group. Ctl, untreated control.

RR-mediated inhibition of NF-*κ*B translocation and NF-*κ*B/I-kB transcription revealed the reason for the marked inhibition of nitrite levels and iNOS/COX-2 expression. This was further confirmed through the measurement of proinflammatory cytokine levels in LPS-activated BV2 microglial cells. NR, RR, and resveratrol demonstrated almost similar potency to inhibit PGE2 secretion, while resveratrol showed the highest potency for inhibition of TNF-*α* and IL-6 production as shown in (Figure 8). The effect of RR for the inhibition of the inflammatory cytokines is significantly higher in comparison to the NR alone as shown in Figure 8, suggesting the better potency of RR to that of NR alone. As resveratrol, the pure compound, and extracts of NR and RR demonstrated almost equipotent ability to inhibit proinflammatory cytokine production, it can be suggested that RR is sufficiently capable to inhibit the production of not only inflammatory mediators but also proinflammatory cytokines.

## 4. Discussion

In this study, we reported the antineuroinflammatory properties of RR for the first time. We compared the cytotoxicity of RR with NR and resveratrol in LPS-stimulated BV2 microglia. In addition, we determined the effects of RR, NR, and resveratrol on the various parameters of neuroinflammation, including COX2, iNOS, nitric oxide, MAPK signaling in both acute and chronic conditions of activated microglia, AP-1 and NF-*κ*B signaling, and inflammatory mediator production in activated microglia. Our findings revealed that RR showed equal or higher anti-inflammatory efficacy than resveratrol alone, without any cytotoxicity.

Neuroinflammation and brain aging leads to continuous degeneration of brain function [32]. Aging process specifically targets the brain, cardiovascular system, and metabolic system, either in intrinsic or extrinsic aging [33]. Aging as well as neuroinflammation is the key mediator of neurodegenerative conditions such as AD and PD [34]. As RR treatment significantly attenuated UVB-induced skin aging, metabolic disorders, and hyperpigmentation both *in vitro* and *in vivo* in our previous study, we further designed an experiment to determine their effect against neuroinflammation, which is a major cause of almost all neurological disorders [28]. Potent inhibition of inflammation by RR treatment in UVB/ROS-induced dermal fibroblasts in our previous report serves as a cue for this experiment [28]. As neuroinflammation caused by overactivated microglia can aggravate neurodegeneration in AD, PD, MS, and ischemic stroke, [35], controlling microglial activation could be a potential strategy for the management of these disorders. Acute but strong activation of microglia is observed in ischemic stroke, while chronic microglial activation and neuroinflammation are observed in AD, PD, and MS [36], indicating that inhibition of microglial activation and its inflammatory cascades is the key therapy against such CNS disorders. Resveratrol, a multifunctional phytochemical, has a proven efficacy against diabetes, cardiovascular disease, obesity, and asthma via alterations in the gut microbiome [37]. Human gut microbiota play an important role in the treatment of various CNS disorders, including neuroinflammation regulation in dementia [38]. It is also possible that the RR-mediated antineuroinflammatory effect might be exerted through the alteration of gut microbiome. Resveratrol at high concentrations might be toxic to the gut microbiome, but RR is completely safe to normal cells as shown in our previous study.

NO production by overactivated microglia is a key biomarker for neuroinflammation in CNS disorders. In a preliminary study, the treatment of NR, RR, and resveratrol showed significant inhibition of nitrite production in LPS-activated BV2 microglial cells, without cellular toxicity. Therefore, we decided to perform a mechanistic study. The inhibition of nitrite production and the IC_50_ value for nitrite release was lower in the RR-treated group than in NR-, resveratrol-, and L-NMMA-treated groups. Increased expression of iNOS is necessary for the increased production and release of NO by activated microglia, whereas COX2 expression is required for its induction of arachidonic acid pathway via increased PGE2 production. In different edible plants, phytochemicals such as sulforaphane, resveratrol, and curcumin have antineuroinflammatory role either through the Sirt1 and NRF2 activation or inhibition of the NF-*κ*B translocation and transcription of the inflammatory proteins [17, 39]. Similarly, NR also possesses the antioxidative, anti-inflammatory phytochemicals such as phenolic acids, flavonoids, anthocyanins, proanthocyanidins, tocopherols, *γ*-oryzanol, and phytic acid [40]. NR contains the *α*-tocopherol, *γ*-tocopherol, and tocotrienol as active ingredients [40, 41] which have the potential to inhibit the cytotoxicity against LPS [42]. In specific, *γ*-tocopherol and its metabolites inhibited the cyclooxygenase activity followed by the inhibition of PGE2 in macrophages and epithelial cells [30]. This finding is also supported by our data that treatment of NR containing *α*-tocopherol and *γ*-tocopherol significantly inhibited the COX-2 expression as well as PGE2 production in acute and chronic microglial activation. In addition, we evaluated the effect of *α*-tocopherol and *γ*-tocopherol and found that treatment of *α*-tocopherol and *γ*-tocopherol not only inhibited the nitrite production but also attenuated the expression of iNOS and COX-2 without cellular toxicity up to the concentration of 1 mg/mL. This data revealed that phytochemicals such as *α*-tocopherol and *γ*-tocopherol in NR were responsible for the anti-inflammatory potential. In addition to this, their presence and their effectiveness/safety profile over resveratrol toxicity make RR a better candidate against neuroinflammation. As inhibition of COX-2 by NR and its phytochemicals is more prominent and COX-2 is involved in the induction of pain [43, 44], the applicability of NR might be highly promising in neuropathic pain model. RR exhibited a minor role in inhibiting COX-2 expression; however, its potency to inhibit iNOS under acute and chronic neuroinflammatory conditions was higher than that of resveratrol and NR. These results collectively support the fact that the higher potency of RR in inhibiting iNOS expression was responsible for its higher potency in inhibiting nitrite production. For a compound or sample to inhibit inflammatory cascades, they must control the inflammatory signaling pathways such as MAPK signaling. This in turn helps in regulating NF-*κ*B translocation, followed by I-kB degradation and AP-1 signaling, and these events facilitate the transcription of inflammatory proteins and proinflammatory cytokines [9, 15]. Treatment of activated microglia with RR significantly inhibited the expression of MAPK proteins as evidenced by attenuated phosphorylation of JNK, ERK, and P38 under acute activation condition. Resveratrol treatment significantly increased the expression of JNK, probably because of its cytotoxic nature to normal cells, while RR downregulated the expression of JNK. These results collectively symbolize the superiority of RR over resveratrol in modulating MAPK signaling. This effect was further confirmed in chronic activation condition. LPS exposure to microglia up to 24 h results in chronic activation and induces continuous cascades of neuroinflammation via inflammatory signaling and inflammatory protein production [45, 46]. RR treatment significantly inhibited the activation of JNK and p38, but it did not show any significant effect on ERK phosphorylation. MAPK signaling takes place within a very short time of toll-like receptor 4 (TLR4) activation; however, in chronic microglial activation, JNK and p38 phosphorylation play imperative roles in inflammatory cascade maintenance and induction [19, 47, 48]. In our study, RR treatment inhibited JNK and p38 phosphorylation with a higher potency than resveratrol treatment. Notably, the potency of NR to inhibit pJNK expression in acute and chronic activation conditions was higher than that of RR and resveratrol alone, which could explain the increase in pJNK expression by resveratrol and significant inhibition of pJNK expression by RR. ERK activation has a dual role: short-term activation occurs during inflammation and long-term activation occurs during cell survival [49]. The increase/insignificant inhibition of pERK expression by NR suggests its role against LPS-induced toxicity by increasing the survival of microglia. Interventions that can control JNK and ERK activation will alter the expression of AP-1 signaling [50, 51]. Resveratrol increased the expression of pJNK and p-C-Jun expression, while RR treatment inhibited the phosphorylation of pJNK and p-C-Jun. Both NR and RR inhibited p-C-Jun and p-C-Fos expression, but resveratrol did not show a similar effect. Furthermore, we assessed the role of RR against NF-*κ*B translocation and I-kB degradation. We observed significantly higher amounts of NF-*κ*B in the cytosol and lower amounts in the nucleus in the RR-treated group, indicating that RR inhibited the translocation of NF-*κ*B from the cytosol to the nucleus. Additionally, significantly low levels of phosphorylated I-kB in the cytosol confirmed that RR inhibited the degradation of I-kB in activated microglia, suggesting the strong antineuroinflammatory effects of RR against activated microglia. Although the resveratrol is a pure compound, we used here to compare the effectiveness of RR (extract of resveratrol-enriched rice) in comparison to NR (extract of normal rice) and resveratrol alone. NR, RR, and R treatment showed the different potencies in different concentrations for inhibition of inflammatory parameters. The highest concentration of resveratrol, i.e., 100 *μ*g/mL, showed the highest potency but it also possessed the cellular toxicity. We found the potential effect of RR that seems to act in synergistic effect of NR and resveratrol. NR also showed good potency to inhibit the inflammatory cascades. Therefore, we hypothesized that the presence of phytochemicals *α*-tocopherol and *γ*-tocopherol in NR can boost the activity of RR either they can help for the protection of cells against resveratrol-mediated cell death or morphological changes. We evaluated the potential antineuroinflammatory effect of *α*-tocopherol and *γ*-tocopherol in rice and found that treatment of *α*-tocopherol and *γ*-tocopherol inhibited not only the nitrite production but also the expression of iNOS and COX-2 without cellular toxicity up to the concentration of 1 mg/mL. This data supports that active phytochemicals including *α*-tocopherol and *γ*-tocopherol in NR are responsible for the anti-inflammatory potential in LPS-treated BV2 cells. Also, we found that the RR-treated group significantly showed the higher potency of RR than resveratrol-treated group alone in experimental group. Hence, the most notable achievement of this study was the improved efficacy of resveratrol following preparation of pharmaceutical crops (rice) containing resveratrol, which showed negligible toxicity on microglial cells.

**Figure 9:**
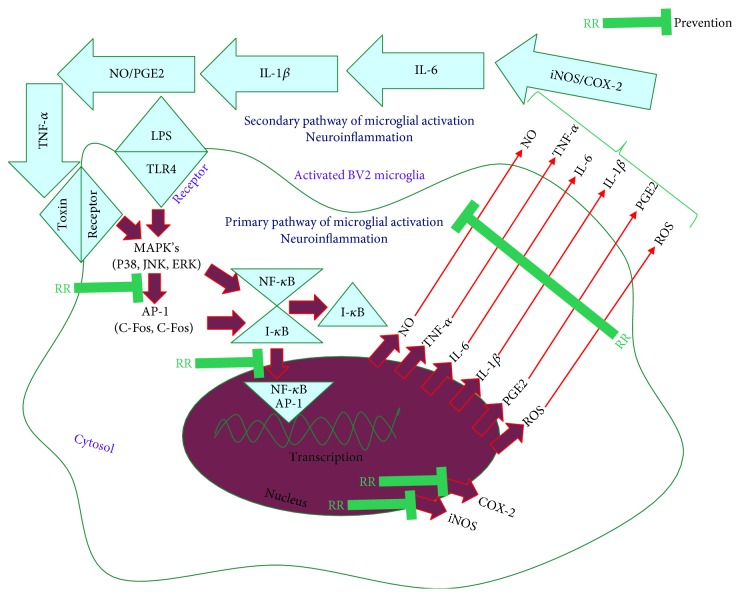
Schematic diagram for the antineuroinflammatory effect of resveratrol-enriched rice (RR) against LPS-induced microglia activation.

In this study, we not only discovered the safe and effective role of RR against aging and neuroinflammation but also identified the antineuroinflammatory potential of NR itself is because of the presence of active phytochemicals such as *α*-tocopherol and *γ*-tocopherol in rice. The anti-inflammatory effect by RR treatment seems to be mediated through inhibition of nitrite production, MAPK phosphorylation, NF-*κ*B mediated production of proinflammatory cytokines, and expressions of inflammatory proteins which are shown in Figure 9 as a summarized figure. Thus, consumption of RR as a functional food will not only act as a nutritional food component but also as a medicinal ambrosia for the prevention and treatment of various human disorders. Further research should be performed to evaluate the antineuroinflammatory potential of RR in various neuroinflammatory disorders through both *in vitro* and *in vivo* studies.

## Data Availability

All the data are included within the manuscript.
